# Supplementary material for: Development of a PET Probe Targeting Bromodomain and Extra-Terminal Proteins for In Vitro and In Vivo Visualization
Source: Pharmaceuticals (Basel). 2024 Dec 11;17(12):1670. doi: 10.3390/ph17121670 (PMC11677465; doi:10.3390/ph17121670)
Supplement: Supplementary file 1 [file pharmaceuticals-17-01670-s001.zip › pharmaceuticals-3355312-supplementary.pdf]

pharmaceuticals

Supplementary Material

A. General information 1

B. Experimental Procedure and Binding Affinity Results for YL10 1–2

C. Purity analysis 2

D. Chemistry Methods 3–5

E. NMR spectrums for the synthesized compound 6

A. General information

We preformed study followed by our previous method. We obtained $[^{11}C]CO_2$ via the 14N (p, α)^{11}C reaction with 2.5% oxygen and 11 MeV protons (Siemens Eclipse cyclotron) in nitrogen, and captured on molecular sieves by TRACERlab, FX-MeI synthesizer (General Electric). We obtained $[^{11}C]CH_4$ by reducing $[^{11}C]CO_2$ in the presence of Ni/hydrogen at 350 °C and recirculated it through an oven equipped with I^2, and produced $[^{11}C]CH_3I$ by radical reaction.

For this study, a total of eight male C57BL6 mice, aged 5 months, were employed. All mice research were carried out at Massachusetts General Hospital (PHS Assurance of Compliance No. A3596-01). The Subcommittee on Research Animal Care (SRAC) serves as the Institutional Animal Care and Use Committee (IACUC) for the Massachusetts General Hospital (MGH). To minimize mice (C57BL/6) discomfort, we performed PET/CT imaging in anesthetized (isoflurane) mice. Throughout the process, animal safety is monitored by trained animal technicians. Daily care is the responsibility of the veterinarian. Keep all mice socially in cages, and give them unlimited food and water, and provide extra nutritional supplements as required by the attending veterinarian.

B. Experimental Procedure and Binding Affinity Results for YL10

Protocol Description

Bromodomain assays. T7 phage strains displaying bromodomains were grown in parallel in 24-well blocks in an E. coli host derived from the BL21 strain. E. coli were grown to log-phase and infected with T7 phage from a frozen stock (multiplicity of infection = 0.4) and incubated with shaking at 32 °C until lysis (90–150 minutes). The lysates were centrifuged (5,000× *g*) and filtered (0.2 μm) to remove cell debris. Streptavidin-coated magnetic beads were treated with biotinylated small molecule or acetylated peptide ligands for 30 minutes at room temperature to generate affinity resins for bromodomain assays. The liganded beads were blocked with excess biotin and washed with blocking buffer (SeaBlock (Pierce), 1 % BSA, 0.05 % Tween 20, 1 mM DTT) to remove unbound ligand

and to reduce nonspecific phage binding. Binding reactions were assembled by combining bromodomains, liganded affinity beads, and test compounds in 1× binding buffer (17% SeaBlock, 0.33× PBS, 0.04% Tween 20, 0.02% BSA, 0.004% Sodium azide, 7.4 mM DTT). Test compounds were prepared as 1000X stocks in 100% DMSO. Kds were determined using an 11-point 3-fold compound dilution series with one DMSO control point. All compounds for Kd measurements are distributed by acoustic transfer (non-contact dispensing) in 100% DMSO. The compounds were then diluted directly into the assays such that the final concentration of DMSO was 0.09%. All reactions performed in polypropylene 384-well plates. Each was a final volume of 0.02 ml. The assay plates were incubated at room temperature with shaking for 1 hour and the affinity beads were washed with wash buffer (1× PBS, 0.05% Tween 20). The beads were then resuspended in elution buffer (1× PBS, 0.05% Tween 20, 2 μM non-biotinylated affinity ligand) and incubated at room temperature with shaking for 30 minutes. The bromodomain concentration in the eluates was measured by qPCR.

Binding Affinity Results

BRD4(1):

Replicate 1: Kd = 0.51 μM

Replicate 2: Kd = 0.45 μM

Average Kd = 0.48 μM

BRD4(2):

Replicate 1: Kd = 1.70μM

Replicate 2: Kd = 1.60 μM

Average Kd = 1.65 μM

C. Purity analysis

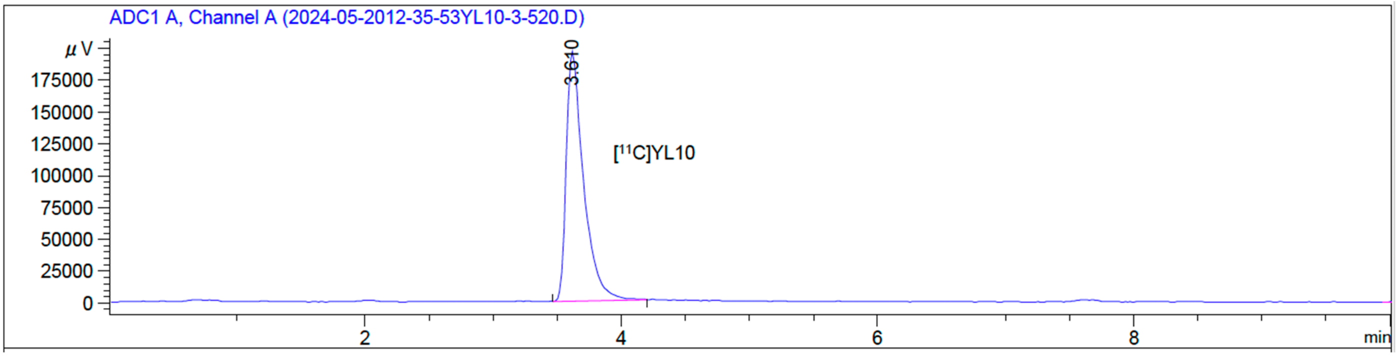

Figure S1. The HPLC chromatogram of [^{11}C]YL10. Analytic HPLC condition: Agilent Eclipse plus C18, 3.5 μm, 4.6 × 100 mm, flow rate = 1.0 mL/min, 0–1 minute: Mobile phase is 0.1% TFA in water/0.1% TFA in acetonitrile, 90/10 (*v*/*v*). 1–9 minutes: Mobile phase is 0.1% TFA in water/0.1% TFA in acetonitrile, Gradient from 90/10 to 10/90 (*v*/*v*). 9–10 minutes: Mobile phase is 0.1% TFA in water/0.1% TFA in acetonitrile, 10/90 (*v*/*v*).

D. Chemistry Methods

All of the commercialized chemical materials were limited to ACSgrade and applied directly. NMR data were obtained on a JEOL Spectrometer(JNM−ECZ500R, ^{1}H 500 MHz, ^{13}C 126 MHz). Chemical shifts were revealed in δ (ppm), taking TMS as the internal standard; coupling constants (J) were given in Hz. Signal multiplicities we redemonstrated by s, d, t.q, m, and br as singlet, doublet, triplet, quartet, multiple, and broad signal, respectively. Column chromato-graphic purification was carried out using silica gel. Agilent 1100 series HPLC was used for analysis and separation. Mass spectrometry data were obtained on an Agilent 1200 series HPLC instrument coupled to an Agilent 6310 mass spectrometer (ESI source).

Synthesis of the Standard Compound YL10 and Precursor YL9

Synthesis of 2-amino-4-bromo-5-methoxy-benzoic acid (2): Methyl 2-amino-4-bromo-5-methoxybenzoate (10 g, 38.45 mmol) was dissolved in 100 mL of tetrahydrofuran (THF). At 20 °C, aqueous lithium hydroxide ($LiOH{\cdot}H_2O$, 2.76 g, 65.83 mmol) was added, and the reaction mixture was stirred at 80 °C for 3 hours until LCMS analysis confirmed the complete consumption of the starting material. The reaction mixture was then acidified to a pH of 5–6 using 6N hydrochloric acid (HCl), followed by filtration. The resulting precipitate, 2-amino-4-bromo-5-methoxybenzoic acid, was obtained as a yellow solid (15.6 g, crude) without further purification. $[M+H]^+$ = 246.0. ^{1}H NMR (DMSO-d_6): δ 3.71 (s, 3H), 7.05 (s, 1H), 7.28 (s, 1H).

Synthesis of 4-bromo-5-methoxy-2-[[(E)-2-nitrovinyl]amino]benzoic acid (3): To a solution of NaOH (4.55 g, 113.79 mmol, 4 eq) in H_2O (30 mL) was added CH_3NO_2 (6.95 g, 113.79 mmol, 6.16 mL, 4 eq) at 0 °C. The resulting solution was stirred at 20 °C for 0.5hr and then at 45 °C for 1hr. Then 2-amino-4-bromo-5-methoxy-benzoic acid (7 g, 28.45 mmol, 1 eq) in HCl (12 M, 15.00 mL, 6.33 eq) was added dropwise to the reaction mixture, which was diluted with ice H_2O (400 mL). The resulting mixture was stirred at 35 °C for 5hr. LCMS showed 75.4% of the peak with desired mass. The mixture was diluted with H_2O(1 L) , filtered and collected precipitates. No further purification was carried out. Compound 4-bromo-5-methoxy-2-[[(E)-2-nitrovinyl]amino]benzoic acid (13.41 g, crude) was obtained as a yellow solid. $[M+H]^+$: 317.0. ^{1}H NMR (400 MHz, DMSO-d_6) δ ppm 3.81–3.93 (m, 3H) 6.71 (d, J = 6.25 Hz, 1H) 7.56 (s, 1H) 7.96–8.18 (m, 2H) 12.88 (br d, J = 13.63 Hz, 1H).

Synthesis of 7-bromo-6-methoxy-3-nitro-quinolin-4-ol (4): The reaction mixture containing 4-bromo-5-methoxy-2-[[(E)-2-nitrovinyl]amino]benzoic acid (1 g, 3.15 mmol) was stirred with acetic anhydride (Ac_2O, 10 mL) and potassium acetate (KOAc, 371.41 mg, 3.78 mmol) at 90 °C for 2 hours. LCMS analysis confirmed the complete consumption of the starting material. The reaction was quenched by adding 30 mL of water, and the resulting solid was filtered, washed with water (3 × 10 mL), and dried under vacuum. This process yielded 7-bromo-6-methoxy-3-nitroquinolin-4-ol (450 mg, 1.17 mmol, 37.05% yield, 77.66% purity) as a black solid. $[M+H]^+$ = 298.8. ^{1}H NMR (400 MHz, DMSO-d_6): δ 3.97 (s, 3H), 7.70 (s, 1H), 7.98 (s, 1H), 9.16 (s, 1H), 12.39–13.60 (m, 1H).

Synthesis of 7-bromo-4-chloro-6-methoxy-3-nitroquinoline (5): A solution of 7-bromo-6-methoxy-3-nitroquinolin-4-ol (450 mg, 1.50 mmol) was prepared in phosphorus oxychloride ($POCl_3$, 1.04 g, 6.77 mmol) and cooled to 0°C under a nitrogen atmosphere. Triethylamine (TEA, 1.39 g, 13.76 mmol) was then added, and the mixture was heated to 100°C, stirring continuously for

5 hours. LCMS confirmed the full consumption of the starting material. After the reaction was quenched with water (5 mL) and acidified with dilute hydrochloric acid (HCl) to pH 5–6, a black solid precipitated. This solid was filtered to give 7-bromo-4-chloro-6-methoxy-3-nitroquinoline (430 mg, 1.29 mmol, 85.55% yield, 95.05% purity) as a black solid. $[M+H]^+$ = 317.0. ^{1}H NMR (DMSO-d_6): δ 4.11 (s, 3H), 7.67 (s, 1H), 8.53 (s, 1H), 9.25 (s, 1H).

Synthesis of (R)-7-bromo-6-methoxy-3-nitro-N-(1-phenylethyl)quinolin-4-amine (6): To a solution of 7-bromo-4-chloro-6-methoxy-3-nitroquinoline (430 mg, 1.35 mmol) and (R)-1-phenylethan-1-amine (164.10 mg, 1.35 mmol) in dimethylformamide (DMF, 1.5 mL), the mixture was stirred at 30°C for 4 hours. LCMS confirmed 72.0% conversion. The reaction was diluted with water, and the black precipitate was filtered to yield (R)-7-bromo-6-methoxy-3-nitro-N-(1-phenylethyl)quinolin-4-amine (330 mg, 755.51 μmol, 55.79% yield, 92.09% purity) as a black solid. $[M+H]^+$ = 402.0. ee%=99.09% (Autopol III – Automatic Polarimeter, Rudolph Research Analytical, USA). ^{1}H NMR (DMSO-d_6): δ 1.69 (br d, J = 6.50 Hz, 3H), 3.60 (s, 3 H), 5.26–5.43 (m, 1H), 7.28 (br s, 1H), 7.35 (br d, J = 7.63 Hz, 2H), 7.46 (br d, J = 7.38 Hz, 2H), 7.56 (br d, J = 3.38 Hz, 1H), 8.10–8.19 (m, 1H), 8.97 (s, 1H), 9.10 (br d, J = 7.25 Hz, 1H).

Synthesis of (R)-7-bromo-6-methoxy-N4-(1-phenylethyl)quinoline-3,4-diamine (7): The mixture containing (R)-7-bromo-6-methoxy-3-nitro-N-(1-phenylethyl)quinolin-4-amine (330 mg, 820.40 μmol), ammonium chloride (NH_4Cl, 197.48 mg, 3.69 mmol), iron powder (Fe, 137.45 mg, 2.46 mmol), ethanol (2 mL), and water (1 mL) was stirred at 90°C under nitrogen for 5 hours. LCMS confirmed complete consumption of the starting material. The mixture was diluted with water (10 mL), filtered, and the filter cake was washed with water. The filtrate was alkalized to pH 8–9 using sodium hydroxide (NaOH). The organic phase was extracted with ethyl acetate (EtOAc, 3 × 5 mL), washed with sodium chloride (NaCl), dried over sodium sulfate (Na_2SO_4), and concentrated to yield (R)-7-bromo-6-methoxy-N4-(1-phenylethyl)quinoline-3,4-diamine (160 mg, 376.17 μmol, 45.85% yield, 89.06% purity) as a yellow solid. $[M+H]^+$ = 372.0. ee%=100% (Autopol III – Automatic Polarimeter, Rudolph Research Analytical, USA). ^{1}H NMR (DMSO-d_6): δ 1.53 (br d, J = 3.38 Hz, 3H), 3.79 (br s, 3H), 4.54 (br d, J = 2.75 Hz, 1H), 5.17 (br s, 2H), 7.09–7.30 (m, 4H), 7.40 (br s, 2H), 7.85 (br s, 1H), 8.24 (br s, 1H).

Synthesis of (R)-7-bromo-8-methoxy-2-methyl-1-(1-phenylethyl)-1H-pyrrolo[3,2-c]quinoline (8): The mixture containing (R)-7-bromo-6-methoxy-3-nitro-N-(1-phenylethyl)quinolin-4-amine (160 mg, 429.81 μmol), 1,1'-carbonyldiimidazole (CDI, 174.23 mg, 1.07 mmol), potassium acetate (KOAc, 84.36 mg, 859.62 μmol), and acetic acid (2 mL) was stirred at 100°C for 5 hours. LCMS confirmed complete consumption of the starting material. The reaction was quenched with ice water (10 mL) and adjusted to pH 8–9 with NaOH. The mixture was extracted with EtOAc (5 × 5 mL). The combined organic extracts were washed with brine, dried over Na_2SO_4, and concentrated to yield (R)-7-bromo-8-methoxy-2-methyl-1-(1-phenylethyl)-1H-pyrrolo[3,2-c]quinoline (120 mg, 212.52 μmol, 49.44% yield, 70.18% purity) as a yellow solid. $[M+H]^+$ = 396.0. ^{1}H NMR (DMSO-d_6): δ 2.00–2.07 (m, 3H), 2.75 (br s, 3H), 3.42 (br s, 3H), 6.34 (br s, 1H), 6.61–6.84 (m, 1H), 7.25 (br d, J = 7.63 Hz, 2H), 7.30–7.41 (m, 3H), 8.28 (s, 1H), 9.06 (s, 1H).

Synthesis of (R)-7-(3,5-dimethyl-1H-pyrazol-4-yl)-8-methoxy-1-(1-phenylethyl)-1,3-dihydro-2H-imidazo[4,5-c]quinolin-2-one (YL9): The reaction mixture consisting of (R)-7-bromo-8-methoxy-2-methyl-1-(1-phenylethyl)-1H-pyrrolo[3,2-c]quinoline (110 mg, 277.58 μmol), 3,5-dimethyl-4-(4,4,5,5-tetramethyl-1,3,2-dioxaborolan-2-yl)-1H-pyrazole (184.95 mg, 832.75 μmol), potassium

acetate (54.49 mg, 555.16 μmol), and Pd(dppf)Cl_2 (40.62 mg, 55.52 μmol) in a mixture of dioxane (3 mL) and water (1 mL) was stirred at 20°C under nitrogen for 20 minutes. It was then heated to 100°C for 2 hours. LCMS indicated 30.9% of the peak with the desired mass. The reaction mixture was concentrated to give a residue. The residue was purified by prep-HPLC (Neutral condition; column: CD07-Daisogel SP-100-8-ODS-PK 150 × 25 × 10 μm; mobile phase: [water (NH_4HCO_3)-ACN]; gradient: 18–48% B over 15 min). Compound (**YL9**) (R)-7-(3,5-dimethyl-1H-pyrazol-4-yl)-8-methoxy-1-(1-phenylethyl)-1,3-dihydro-2H-imidazo[4,5-c]quinolin-2-one (21 mg, 49.38 μmol, 17.79% yield, 96.76% purity) as a white solid. $[M+H]^+$ = 412.2. ee%=100% (Autopol III – Automatic Polarimeter, Rudolph Research Analytical, USA). ^{1}H NMR (DMSO-d_6): δ 1.94–2.12 (m, 10H), 2.75 (br s, 3H), 3.30 (br s, 2H), 6.09–6.49 (m, 1H), 6.55–6.90 (m, 1H), 7.29 (d, J = 7.88 Hz, 2 H), 7.32–7.37 (m, 1H), 7.38–7.44 (m, 2H), 7.74 (s, 1H), 9.01 (s, 1H), 12.25 (br s, 1H).

Synthesis of (R)-8-methoxy-2-methyl-1-(1-phenylethyl)-7-(1,3,5-trimethyl-1H-pyrazol-4-yl)-1H-imidazo[4,5-c]quinoline (YL10): To a solution of YL9 (82.2 mg, 0.2 mmol, 1 eq) in tetrahydrofuran (THF, 2 mL), sodium hydride (NaH, 25.9 mg, 0.65 mmol, 3.2 eq) was added under an ice bath. The mixture was stirred at 0°C for 15 minutes. Subsequently, methyl iodide (CH_3I, 212 μL, 0.34 mmol, 1.7 eq) was added. The reaction was allowed to stir at room temperature (rt) for overnight. Methanol (MeOH) was then added to quench the reaction. The reaction mixture was concentrated, and the residue was purified by prep-HPLC (Neutral condition; column: CD07-Daisogel SP-100-8-ODS-PK 150*25*10um;mobile phase: [water(NH_4HCO_3)-ACN];gradient:18%-48% B over 15 min) to yield the final product, (R)-8-methoxy-2-methyl-1-(1-phenylethyl)-7-(1,3,5-trimethyl-1H-pyrazol-4-yl)-1H-imidazo[4,5-c]quinoline (52.3 mg, 61.5% yield, 97.68% purity by LCMS) as a white solid. $[M+H]^+$ 426.1. ee%=100% (Autopol III – Automatic Polarimeter, Rudolph Research Analytical, USA). ^{1}H NMR (500 MHz, CHLOROFORM-D) δ 9.18 (s, 1H), 7.96 (s, 1H), 7.39 (d, J = 6.8 Hz, 1H), 7.40–7.31 (m, 2H), 7.26 (d, J = 7.6 Hz, 2H), 6.71 (s, 1H), 6.17 (s, 1H), 3.76 (s, 3H), 3.29 (s, 3H), 2.78 (s, 3H), 2.12 (dd, J = 10.2, 8.6 Hz, 9H).

Radiosynthesis of [^{11}C]YL10: The radiosynthesis of [^{11}C]YL10 was performed using a conventional methylation technique. Illustrated in Scheme 2, the process began by trapping [^{11}C]CH_3I in anhydrous DMF (300 μL), along with precursor compound YL9 (1.0 mg) and KOH (3.0 mg). The reaction mixture was heated to 100°C for 3 minutes. To quench the radioactive reaction, HPLC mobile phase (0.5 mL) was added, and the compound was isolated via reverse-phase semi-preparative HPLC (Agilent Eclipse XDB-C18, 5 mm, 250 mm × 9.4 mm, flow rate = 5.0 mL/min, mobile phase is 0.1% TFA in water/0.1% TFA in acetonitrile, 60/40, *v*/*v*), yielding 21–29% (decay-corrected from trapped [^{11}C]CH_3I). The [^{11}C]YL10, identified by a retention time of 10.5 minutes, was diluted in water (15 mL) and passed through an SPE C-18 cartridge. After rinsing with water (10 mL), the product was eluted using ethanol (1.5 mL). The procedure ensured a radiochemical purity exceeding 95% by HPLC(Agilent Eclipse plus C18, 3.5 μm, 4.6×100 mm, flow rate = 1.0 mL/min, 0–1 minute: Mobile phase is 0.1% TFA in water/0.1% TFA in acetonitrile, 90/10 (*v*/*v*). 1–9 minutes: Mobile phase is 0.1% TFA in water/0.1% TFA in acetonitrile, Gradient from 90/10 to 10/90 (*v*/*v*). 9–10 minutes: Mobile phase is 0.1% TFA in water/0.1% TFA in acetonitrile, 10/90 (*v*/*v*).). The identity of [^{11}C]YL10 was confirmed by co-injection with a non-radioactive YL10 standard, and the final [^{11}C]YL10 solution was prepared in sterile saline (2.7 mL) for subsequent in vivo studies. The enantiomeric excess (ee%) of [^{11}C]YL10, measured after decay, was determined to be 100%, confirming its enantiomeric purity. The measurement was conducted using an Autopol III Automatic Polarimeter (Rudolph Research Analytical, USA).

E. NMR spectrums for YL10

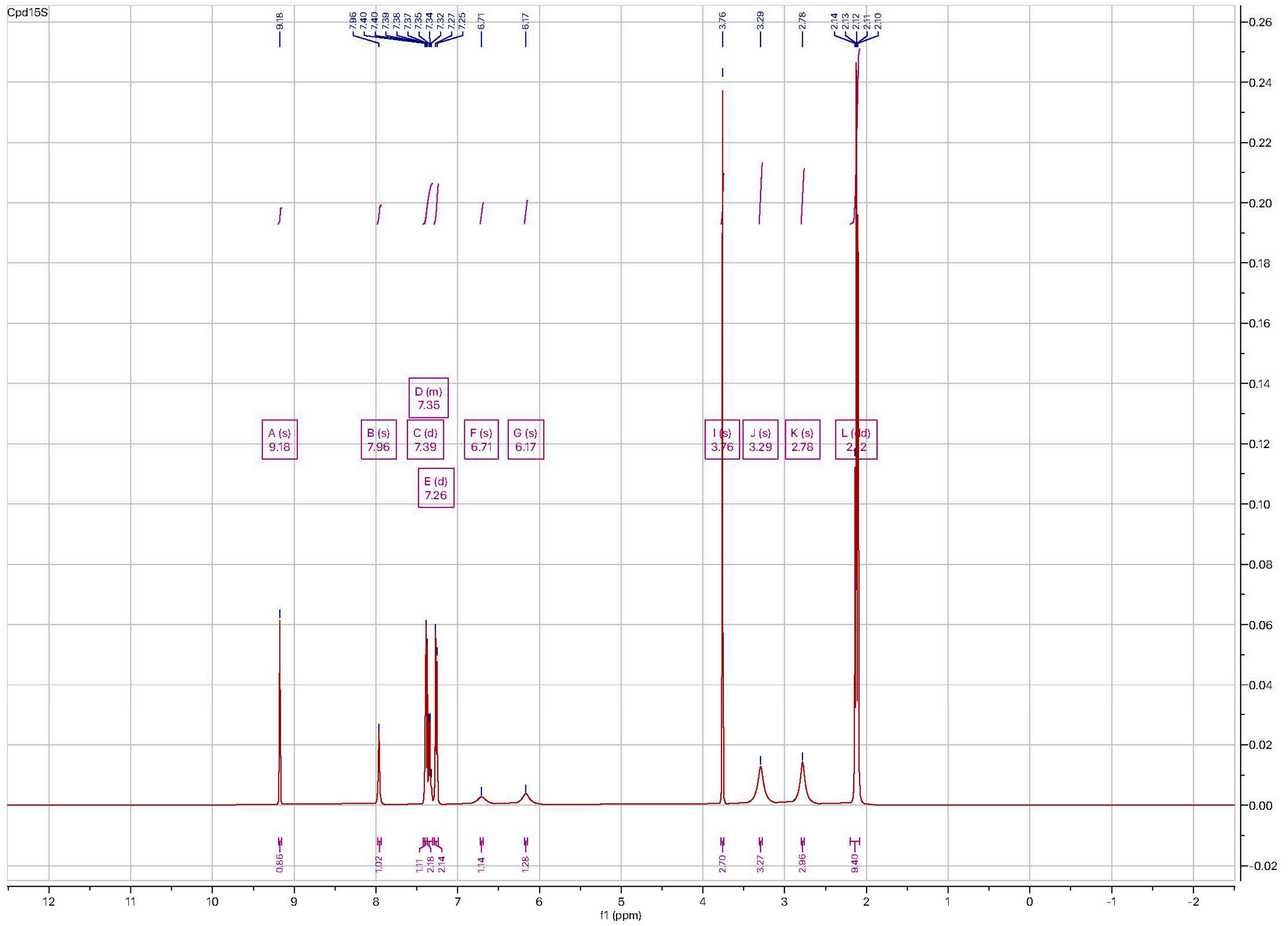

Figure S2. NMR spectrums for YL10
